# Development of a Synbiotic Beverage Enriched with Bifidobacteria Strains and Fortified with Whey Proteins

**DOI:** 10.3389/fmicb.2017.00640

**Published:** 2017-04-19

**Authors:** Federico Baruzzi, Silvia de Candia, Laura Quintieri, Leonardo Caputo, Francesca De Leo

**Affiliations:** ^1^Institute of Sciences of Food Production, National Research Council of Italy (ISPA-CNR)Bari, Italy; ^2^Institute of Biomembranes, Bioenergetic and Molecular Biotechnologies, National Research Council of Italy (IBIOM-CNR)Bari, Italy

**Keywords:** *Bifidobacterium*, inulin, resistant starch, whey proteins, fortified beverage

## Abstract

The objective of this study was to develop a new synbiotic beverage evaluating the ability of some bifidobacteria strains to grow in this beverage which was fortified with whey proteins up to 20 g L^-1^, and enriched with 10 g L^-1^ of prebiotic inulin or resistant starch. The ability of *Bifidobacterium* strains to survive for 30 days at 4°C was evaluated in two synbiotic whey protein fortified beverages formulated with 2% of whey proteins and 1% of inulin or resistant starch. Microbial growth was significantly affected by the whey protein amount as well as by the kind of prebiotic fiber. Resistant starch promoted the growth of the *Bifidobacterium pseudocatenulatum* strain and its viability under cold storage, also conferring higher sensory scores. The development of this new functional beverage will allow to carry out *in vivo* trials in order to validate its pre- and probiotic effects.

## Introduction

Functional foods are defined as foods or food ingredients that may provide a health benefit beyond the basic nutrition (IOM/NAS, 1994; Gibson and Williams, 2000). In the last century, a huge amount of research describes the role of *Bifidobacterium* genus in promoting human and animal health (Preising et al., 2010; Fukuda et al., 2011; Russell et al., 2011). Bifidobacteria have been also proposed to promote the correct microbial equilibrium of the intestinal microbiota in preterm infants (Szajewska et al., 2010) due to their beneficial effects on human health and predominance in the intestinal microflora. Furthermore, it has been demonstrated that the consumption of indigestible carbohydrates, such as resistant starch (RS), can enhance positive biological effects induced by natural occurring bifidobacteria in the human gastrointestinal tract and usually defined as prebiosis (Saulnier et al., 2009).

Indeed, bifidobacteria, exploiting their unique glycosidases, transporters, and metabolic enzymes for sugar fermentation, are able to activate fermentable molecules in an environment poor in nutrition and oxygen (Fushinobu, 2010).

Resistant starch, naturally present in many vegetables (e.g., plantains, green bananas, roots, and legumes) or obtained from processed cereals (Quintieri et al., 2012b), withstand the upper gastrointestinal digestive enzymes; in the distal colon, RS, can be preferentially and specifically hydrolyzed by bifidobacteria displaying bifidogenic effects (Nugent, 2005; Queiroz-Monici et al., 2005; Regmi et al., 2011).

Inulin, a linear D-fructose polymer containing small amounts of branched fructose by β(2-1)-glycosidic bonds with a terminal glucose moiety, is naturally present in several plants (Van Loo et al., 1995); it is partially fermented in the distal colon by bifidobacteria with a consequent increase in their population (De Vuyst and Leroy, 2011).

The microbial fermentation of both inulin and RS in the colon produces short chain fatty acids (SCFA; acetate, propionate, and butyrate, lactate), succinate, hydrogen and carbon dioxide; these metabolites, and in particular butyrate, are considered to improve water absorption in the large bowel, to modulate colonic muscular activity, to inhibit the growth of cancer cells, to stimulate the growth of normal cells, and to promote DNA repair in damaged cells, as recently reviewed by Topping et al. (2008). Based on these studies, World Health Organization (WHO) recommends to supplement the diet with low-carb fiber-rich meals in order to maximize colonic disease prevention and reduce body weight (WHO, 2015); thus, inulin and RS are commonly used to enrich different kinds of foods (Crittenden R.G. et al., 2001; Brown, 2004; Topping et al., 2008; Fushinobu, 2010; Pokusaeva et al., 2011). In addition to indigestible fibers, milk proteins also showed bifidogenic effects. In particular, α-lactalbumin was found to promote the growth of *Bifidobacterium breve, Bifidobacterium bifidum, Bifidobacterium infantis*, as well as caseinomacropeptide, isolated from whey protein concentrates, and supplemented in milk was reported to increase *Bifidobacterium lactis* counts in probiotic fermented milks (Pihlanto-Leppälä et al., 1999; Janer et al., 2004). The hydrolysates of whey proteins also boosted the growth of *Bifidobacterium animalis* subsp. *lactis* BB12 in yogurt preparation (Tian et al., 2015). Moreover, whey protein hydrolysates are rich in peptides endowed with hypotensive, anticancer, opioid antagonistic, and immunomodulatory properties (Morris and Fitzgerald, 2009). Antimicrobial peptides released from many whey proteins (Naidu, 2000) were also found useful for improving food safety and quality (Quintieri et al., 2012a, 2013; Baruzzi et al., 2015; Caputo et al., 2015). The nutritional and physiological aspects of whey protein based foods are well known as previously reported (Walzem et al., 2002; Morris and Fitzgerald, 2009) and, due to their high amount of digestible proteins and branched amino acids, they are usually consumed by sportsmen to strengthen the muscle anabolism (Walzem et al., 2002; Tang et al., 2009; Pennings et al., 2011; Deutz et al., 2014). Moreover, whey proteins could be also useful for debilitated subjects or people under restrictive diets (such as elderly people, gastrectomized or immunocompromised patients). The consumer demand for a daily intake of fibers and molecules with high nutritional value (as reported by WHO, 2003; National Health and Medical Research Council [NHMRC] and New Zealand Ministry of Health, 2006; Fungwe et al., 2007; EFSA Panel on Dietetic Products, Nutrition, and Allergies (NDA), 2010) has driven this work toward the optimization of a beverage endowed with these features.

Therefore, in this work three bifidobacteria strains (*B. animalis* subsp. *lactis* BI1 and BB12 and *B. breve* BBR8) also applied from the production of probiotic dairy foods (such as BB12) and the *Bifidobacterium pseudocatenulatum* M115 endowed with promising pro-healthy features were chosen in order to develop a new synbiotic beverage. Firstly, the influence of inulin and RS on the viability of bifidobacteria in a fermented whey protein medium was evaluated under laboratory conditions. Then, a synbiotic whey protein beverage enriched with prebiotic fibers was set up and characterized for microbial viability, residual fiber content, and organoleptic properties throughout cold storage period.

## Materials and Methods

**FIGURE 1:**
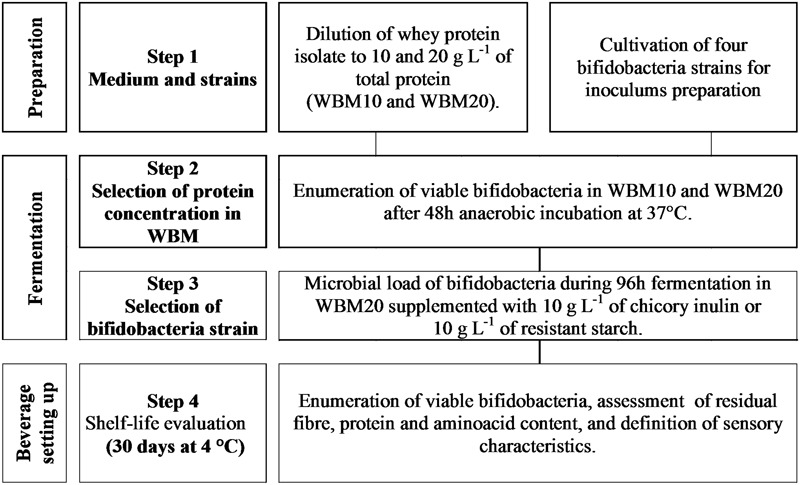
**Graphical scheme of the experimental plan carried out in the present work**.

Experimental plan was arranged on three levels (**Figure 1**) that included the optimization of a whey based medium (WBM) able to sustain bifidobacteria growth (1), the selection of bifidobacteria strains fermenting fibers in WBM (2), then the set up of the synbiotic beverage (3) which was evaluated for its shelf life and sensory profile over 30 days of cold storage.

### Microorganisms and Media

The strains *B. animalis* subsp. *lactis* BI1 and *B. breve* BBR8 were a gift of the Centro Sperimentale del Latte S.r.l. (Lodi, Italy), the *B. pseudocatenulatum* M115 was from the Dairy Research Institute of Asturias (IPLA, Villaviciosa, Spain, Department of Science and Food Technology of the Spanish National Research Council, CSIC) and *B. animalis* subsp. *lactis* BB12 was previously isolated and included in the ISPA-CNR bacterial collection.

Fresh microbial cultures of bifidobacteria strains from frozen cultures (-80°C) were routinely grown in MRS (MRS Agar ISO Formulation, Biolife Italiana srl, Milan, Italy) amended with 0.5 g L^-1^ of L-cysteine (MRSC) for 48h at 37°C under anaerobic conditions (ANAEROGEN, AN0025, Oxoid S.p.A., Milan, Italy).

### Growth of Bifidobacterium Strains in Whey-Based Medium (WBM)

Bifidobacteria strains were evaluated for their ability to grow in whey-based media (WBM). Whey protein isolate (WPI, Mirabol^®^ Whey Protein Natural 97, Volchem s.r.l., Grossa di Gazzo, Italy), containing *ca.* 97 g of whey proteins on 100 g of powder, was diluted in distilled water in order to obtain two WBM at 10 and 20 g L^-1^ of total protein (WBM10 and WBM20). Based on Mirabol^®^’s nutritional fact, the raw composition per liter of WBM10 included 5 g of β-lactoglobulin, 1.7 g of α-lactalbumin, 1.8 g of other whey proteins, 0.02 g of simple sugars, 0.04 g of lipids; the protein concentration in WBM10 allowed to estimate a content 2.3 and 0.5 g L^-1^ of branched chain and sulfur-containing amino acids, respectively. In the case of WBM20 the concentration of nutrients doubled. In addition, WBM were supplemented with 0.5 g L^-1^ of L-cysteine and 10 g L^-1^ of lactose. Fresh bifidobacteria cultures were diluted in sterile saline solution in order to read an absorbance at 600 nm of 0.4 ± 0.05 (*ca.* 8 log cfu mL^-1^), further diluted 1000 times in WBM10 and WBM20 and incubated as above described; MRSC was used as positive control. Then, bifidobacteria were enumerated on Bifidus Selective Medium (BSM) agar plates (Sigma–Aldrich SRL, Milan, Italy) recording pink-purple colored colonies. WBMs were also enriched with prebiotic fiber by the addition of 10 g L^-1^ of chicory inulin with a degree of polymerization > 23 (Orafti HP, BENEO-Orafti, Tienen, Belgium) or 10 g L^-1^ of retrograded RS (Novelose^®^ 330, Ingredion Incorporated, Westchester, IL, USA) and then pasteurized at 110°C × 5 min. In order to reduce whey protein precipitation, thermal treatment parameters were set up by preliminary assays; however, the efficacy of pasteurization was preserved. Prebiotic WBMs were cooled at room temperature and then inoculated with fresh cultures of bifidobacteria, as described above. Strains were incubated under anaerobic conditions at 37°C up to 96 h. Viable cell evaluations were carried out in triplicate every 24 h, as described above.

### Set Up of a Whey Protein Fortified Beverage Enriched with Prebiotic Fibers (WPF Beverage)

Two synbiotic whey protein fortified beverages enriched with prebiotic fiber (500 ml) were obtained, in triplicate, by supplementing WBM, at the protein concentration selected in the section “Growth of Bifidobacterium Strains in Whey-based Medium (WBM),” with inulin or with RS (1%). Glass bottles were filled with WPF beverages leaving less than 5 ml empty space on the top of the fluid, under the cork. After pasteurization and cooling, WPF beverages were inoculated with the bifidobacteria strains M115 or BBR8 (**Figure 1**, Step 3) at approximately 4 log cfu mL^-1^ by using a fresh bifidobacteria culture.

WPF beverages were incubated at 37°C under anaerobic conditions in order to obtain a concentration of bifidobacteria of at least 8 log cfu mL^-1^; depending on the results achieved in the fermentation assays, the incubation ranged from 48 to 72 h. After fermentation, WPF beverages were refrigerated at 4°C for 30 days. WPF beverages without bifidobacteria were also prepared as negative controls.

#### Chemical and Microbiological Analyses of WPF Beverage

During cold storage (at days 0, 15, and 30), each WPF beverage was evaluated for pH (Model pH50 Lab pHMeter XS-Instrument, Concordia, Italy), concentration of viable bifidobacteria, residual fiber, protein and aminoacid content, and sensory characteristics. The percentage of resistant starches and inulin were determined with the KR-STAR kit and with the K-FRUCHK kit (Megazyme International Ltd., Wicklow, Ireland), respectively, following the manufacturer’s instructions.

Protein concentration was determined by the Bradford Method whereas small peptide and aminoacid concentrations were determined after derivatization with *o*-phthaldialdehyde as previously described (Baruzzi et al., 2012).

#### Sensory Evaluation of WPF Beverage

Sensory evaluation was performed recruiting three groups of five habitual consumers of milk beverages. The samples (30 mL) of the different beverages, stored at 4°C for 0, 15, and 30 days, were served at 12 + 1°C in white plastic cups coded with random three-digit numbers, and mineral water at room temperature was provided for mouth-rising. The overall acceptability of each sample was calculated using a 9-point hedonic scale ranging from 1 (“dislike extremely”) to 9 (“like extremely”) and the level of suitability of taste, sweetness, milky appearance, flavor, and mouthfeel, using a 5-point just about right (JAR) scale (1 = too weak, 3 = just about right; 5 = too strong), was followed for each sample, according to Villegas et al. (2010).

### Statistical Analysis

The concentration of viable cells in samples was calculated as the average number of colonies found for each decimal dilution, corrected by the dilution factor and expressed as the log cfu mL^-1^ ± standard deviation. Bifidobacteria viable cell counts, RS and inulin content were analyzed by one-way analysis of variance (ANOVA) carried out using IBM SPSS Statistics 22 (IBM Corporation, Armonk, NY, USA). Tukey’s test and Fisher LSD *post hoc* test were applied in order to evaluate significant differences (*P* ≤ 0.05) among means of viable cell counts and dietary fibers, respectively. In order to compare inoculum levels as well as viable cell load recorded after the same period of incubation among different experiments, the Mann–Whitney non-parametric test was applied.

The independent effects and interactions of the main factors (times of storage, beverages and sensory attributes) on sensory scores were evaluated applying a three- and two-way ANOVA (*P* ≤ 0.05); multiple comparisons among individual means of the same strain were made by Fisher’s LSD *post hoc* test after rejecting the homogeneity of their variances with the Levene’s test (*P* ≤ 0.05).

## Results and Discussion

### Effect of Whey Proteins on Bifidobacteria Growth

The viable cell counts of Bifidobacteria, grown for 48 h at 37°C, in MRSC and in WBM supplemented with different amount of whey proteins (10 or 20 g L^-1^; WBM10, WBM20), were reported in **Table 1**. WBM20 cultures of M115, BBR8, and BI1 showed cell counts significantly higher (*P* < 0.05) than those retrieved in WBM10, although all cultures reached the highest load values in MRSC (9.08 log cfu mL^-1^ on average). This result was in accordance with preliminary experiments carried out using different amounts of whey protein concentrate at 80% of proteins (Baruzzi et al., 2014). Furthermore, BB12 cell counts (7.75 log cfu mL^-1^, on average) in WBM10 and WBM20 were consistent with results of Matijević et al. (2008), who also reported no significant difference in cultures of this strain grown in medium containing 1.5 or 3% of whey protein concentrate. On the basis of these results, the 20 g L^-1^ concentration was chosen for the subsequent analyses.

**Table 1:** Viable cell counts (mean values ± standard deviation of three independent experiments, expressed as log cfu mL^-1^) of *Bifidobacterium* strains in MRSC and WBM at 10 and 20 g L^-1^ protein concentration after 48h of anaerobic fermentation at 37°C.

Strain	MRSC	WBM10	WBM20
*B. animalis* ssp. *lactis* BB12	9.37 ± 0.38^a^	7.40 ± 0.27^b^	8.09 ± 0.27^b^
*B. animalis* ssp. *lactis* BI1	8.61 ± 0.34^a^	4.37 ± 0.31^c^	6.73 ± 0.33^b^
*B. breve* BBR8	9.55 ± 0.08^a^	4.65 ± 0.19^c^	7.43 ± 0.22^b^
*B. pseudocatenulatum* M115	8.80 ± 0.73^a^	4.50 ± 0.46^c^	7.40 ± 0.38^b^

*Initial average microbial log cfu mL^-*1*^ 4.57 ± 0.05. Different letters indicate significant differences (*P* < 0.05) for a least significant difference (LSD) of: BB12, 0.97; BI1, 1.03; BBR8, 0.56; M115, 1.69.*

### Effect of Dietary Fibers on Bifidobacteria Growth

Aimed at promoting bifidobacteria growth at the similar levels of MRSC, used as a reference medium, within the same period of incubation, WBM enriched with total whey proteins to 20 g L^-1^ (WBM20) was amended with additional carbon sources different from lactose.

**FIGURE 2:**
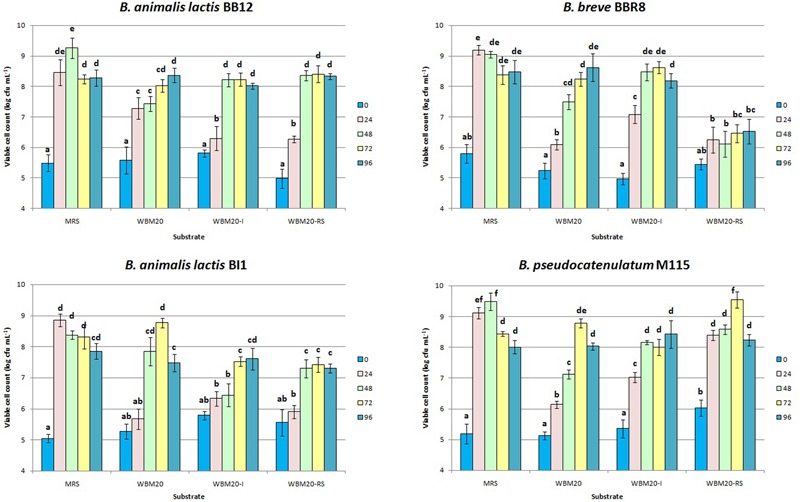
**Viable cell concentration of bifidobacteria strains throughout 96 h of anaerobic incubation at 37°C in MRS and in medium containing 20 g L^-1^ of whey proteins (WBM20), in WBM20 supplemented with 10 g L^-1^ of chicory inulin (WBM20-I) or 10 g L^-1^ of resistant starch (WBM20-RS).** Bars represent mean value of viable cell count of three independent experiments (± standard deviation); values with different lowercase letters are significantly different (*P* < 0.05) according Tukey’s test, within the same strain comparison.

Therefore, the WBM20 was supplemented with 10 g L^-1^ of inulin (WBM20-I) or RS (WBM20-RS), as already assayed by Crittenden R. et al. (2001) and Rossi et al. (2005). Inulin and RS were selected as indigestible for humans but favor bifidobacteria growth. **Figure 2** showed that the addition of 10 g L^-1^ of inulin or the addition of 10 g L^-1^ of RS to WBM20 (containing 10 g L^-1^ of lactose) allowed to obtain microbial growth values similar to those occurred in MRS containing 20 g L^-1^ of glucose only when time of incubation was extended.

However, bifidobacteria growth kinetics demonstrated to be different after WBM20 was supplemented with inulin or RS; in particular, BI1 was negatively affected by both probiotic fibers, whilst M115 was favored by RS. Inulin caused a significant and faster growth increase of both M115 and BBR8 strains. Mann–Whitney analysis also showed the viable cell concentrations of all strains at T0 and T48 throughout the experiments of steps 2 and 3 were similar (*P* > 0.05) at experimental steps 2 and 3 (**Table 1** and **Figure 1**).

In accordance with previous studies, our results showed the bifidobacteria growth in presence of glucose (herein included in MRS) or other monosaccharides was improved in presence of complex carbohydrates (Palframan et al., 2003; Rossi et al., 2005; Rada et al., 2008).

Since amylolytic activity is present in approximately 60% of bifidobacteria species (Crittenden R. et al., 2001) only BB12 and M115 strains increased their viable cell loads after the addition of 10 g L^-1^ of RS to WBM20. This finding agrees with Wronkowska et al. (2006) demonstrating either a good growth and an acidifying activity of bifidobacteria in fermented heat-treated starch. With regards to inulin supplementation, the higher cell loads were found only for the M115 and BBR8 strains in WBM20-I, compared to WBM20, and results were in accordance with Selak et al. (2016) reporting the heterogeneously inulin hydrolysis spread among Bifidobacteria species.

Moreover, in this work the growth of *B. pseudocatenulatum* M115 was favored by inulin-type fructans at each time of sampling over 72 h incubation suggesting the presence and expression of β-fructofuranosidase genes as already reported (Janer et al., 2004; Omori et al., 2010). In addition, M115 was the only strain able to grow better also in presence of RS; this behavior could be attributed to the occurrence of glycosyl transferase, starch, and glycoside hydrolase genes generally found in the genome of bifidobacteria (Liu et al., 2015) and particularly in the genome of the probiotic strain *B. pseudocatenulatum* IPLA 36007 (Alegría et al., 2014). These results increase the knowledge about the *B. pseudocatenulatum* M115 already proposed as a powerful probiotic strain thanks to its interesting pro-healthy features (Delgado et al., 2008; Losurdo et al., 2013).

### Effect of Cold Storage on Viability of Bifidobacteria in WPF Beverages

In the light of previous results, the strain BI1 was excluded as both fibers reduced its viable cell load, conversely, the BBR8 was considered for beverage production only when WBM20 was supplemented with inulin as this fiber promoted its growth within 48 h.

As concerns BB12 and M115, the viable cell loads of both strains increased in WBM20-RS in comparison with WBM20 after 48 and 72 h, respectively. However, due to higher value reached by M115, this strain was selected and BB12 was excluded.

**FIGURE 3:**
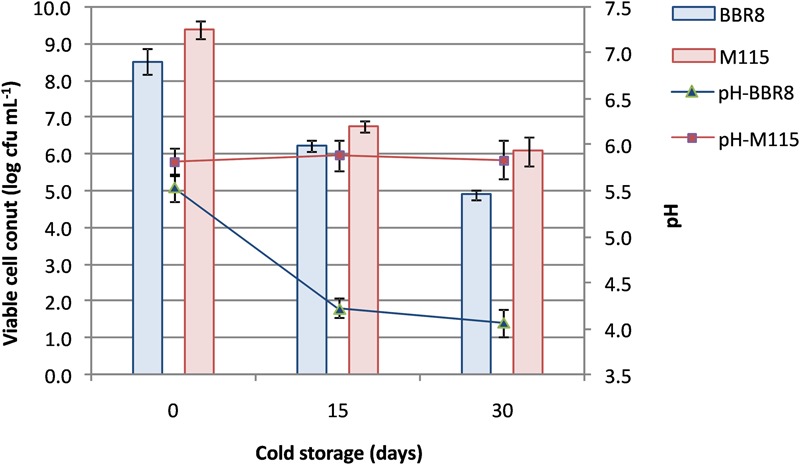
**Changes in pH values and viability of *Bifidobacterium breve* BBR8 and *B. pseudocatenulatum* M115 in whey beverages supplemented with inulin and resistant starch, respectively, throughout 30 days of cold storage**.

Thus, two synbiotic beverages fortified with whey proteins (at 20 g L^-1^; WPF) were manufactured: the first beverage contained inulin and the strain BBR8, whilst the second one contained RS and it was fermented with the strain M115. After fermentation (48 h for the BBR8 and 72 h for the M115), WPF beverages were stored at 4°C evaluating viable cell count and pH (**Figure 3**) until day 30.

Confirming the results of preliminary fermentation assays, bifidobacteria reached a load by means of 8.5 log cfu mL^-1^; then a reduction of *ca*. 2 log cycles was found in beverages within 15 days of cold storage. Subsequently, cell concentration remained stable for *B. pseudocatenulatum* M115, whereas a further reduction was observed in *B. breve* BBR8 reaching *ca.* 4.9 log cfu mL^-1^ (**Figure 3**).

The fiber concentration in WPF beverages during 30 days of cold storage is reported in **Table 2**.

**Table 2:** Fiber content in WPF beverages calculated before and after fermentation and throughout 30 days of cold storage.

	Inulin (g L^-1^)^1^	RS (g L^-1^)^2^
Pre-pasteurization	6.77 ± 0.36^a^	6.53 ± 1.08
Post-pasteurization	5.77 ± 0.53^ab^	5.10 ± 0.83
T0f	6.52 ± 0.42^a^	6.73 ± 0.75
T48f/T0cs	4.74 ± 0.43^b^	5.46 ± 1.99
T15cs	3.24 ± 0.51^c^	5.16 ± 0.94
T30cs	2.65 ± 0.43^c^	7.26 ± 1.55

*f, fermentation; cs, cold storage. Different letters represent average values statistically different (*P* < 0.05) according to Tukey’s test within column. ^*1*^WPF beverage fermented with *B. breve* BBR8. ^2^WPF beverage fermented with *B. pseudocatenulatum* M115.*

In the case of inulin, after its supplementation in WPF beverage, its concentration was 6.77 ± 0.36 g L^-1^, far below the estimated amount reported on the product label (10 g L^-1^ of Orafti^®^ HP, corresponding to *ca*. 9.9 g of pure inulin); this difference could be attributed to the different methods used to determine fructans, AOAC 997.08, as stated by the BENEO-Orafti, and the AOAC 999.03 of the Megazyme kit used in this work. At the end of cold storage inulin concentration halved (**Table 2**).

At contrary, the amount of RS remained quite stable (*ca*. 6 g L^-1^) during both the manufacturing process and the first 15 days of cold storage (**Table 2**). The large standard deviation found in the concentration of RS could be ascribed to their low solubility in water phase; in addition, as reported by the K-RSTAR kit manufacturers, higher errors are expected for samples with RS content lower than 2%.

Since the concentration of RS did not change during preparation, fermentation, and cold storage, it is possible to argue that the growth improvement recorded for M115 (**Figure 2**) could be due to non-resistant polysaccharides supplied with Novelose^®^ 330.

At the end of fermentation, the total protein content of BBR8- or M115-fermented beverages, did not change significantly (*P* < 0.05) remaining stable at their initial average value of 4.19 ± 0.13 mg mL^-1^. Conversely, the concentrations of small peptides and amino acids dropped from 161.6 ± 4.8 (control samples) to 11.99 ± 0.36 and 8.58 ± 0.26 μg mL^-1^ for BBR8 and M115 strains, respectively. No changes in total protein content as well as in small peptide and amino acid contents were found in both WPF beverages during cold storage (data not shown).

As concerns the reduction of viability of BBR8 and MM115 strains during refrigerate period, our result agreed with those of Donkor et al. (2006) and Jayamanne and Adams (2009) reporting the reduction in viable cells of *B. lactis* LAFTI^®^ B94 or of *B. longum* and *B. animalis* ssp. *lactis* strains in yogurt over cold storage, respectively. Part of this decrease could be also attributed to the absence of L-cysteine that did not protect bifidobacteria cells against deleterious effects of cold storage, as demonstrated in refrigerated milk by Bolduc et al. (2006). We removed L-cysteine to avoid a negative influence on flavor. Refrigeration temperature hampered the increase in microbial load; in addition, cell viability was negatively affected by the post-acidification phenomenon as recently reported for six bifidobacteria cold stored for 21 days in milk (Djelled et al., 2016).

In our beverages the survival of bifidobacteria reached the same levels reported by Kailasapathy (2006) after cell microencapsulation, and was higher than that found by Antunes et al. (2005) in a probiotic yogurt containing a *B. longum* strain and enriched with both whey protein concentrate and skim milk powder. It is possible to argue that the occurrence of fibers in WPF beverages helped the survival of bifidobacteria as demonstrated by Crittenden R.G. et al. (2001) in yogurt supplemented with both RS and inulin, throughout 5 weeks of cold storage period. The inulin supplementation (40 mg g^-1^ of reconstituted skim milk) has been already reported by de Souza Oliveira et al. (2011, 2012), to cause an increase in the viable cell load of a *B. animalis* subsp. *lactis* strain or in the biomass of lactic acid bacteria; however, no data were produced about potential inulin consumption. The decrease in the inulin content found during 30 days of cold storage (to about 40% of the initial amount) suggests that inulin was consumed by the strain. The reduction in the inulin content together with the drop in pH values could be consistent with the production of SCFA from this fiber as already demonstrated either by single strains (Marx et al., 2000) and fecal cultures (Pompei et al., 2008; De Vuyst and Leroy, 2011).

The bifidogenic effect of RS has been widely reported (Wang et al., 2002; Bouhnik et al., 2004; Lesmes et al., 2008); however, to the best of our knowledge, no studies were reported about the effect of RS on the growth of bifidobacteria monocolture and their fate in milk-based beverage under the experimental conditions reported in this work. It is interesting to note that the M115 strain reached a viable cell load in WPF amended with RS close to that found in different milk based foods and considered sufficient to provide therapeutic benefits (Shin et al., 2000; Pérez-Conesa et al., 2005). Differently from the WPF beverage amended with inulin, the stable content of RS during storage suggests that this WPF beverage could contribute to the adult dietary fiber daily intake.

As concerns protein content, the results of this work confirm the well known low proteolytic activity of *Bifidobacterium* strains, in particular when compared with that of lactic acid bacteria (Klaver et al., 1993), that in our case assimilated preferentially free amino acids for their metabolisms during fermentation.

### Sensory Evaluation of WPF Beverages

**FIGURE 4:**
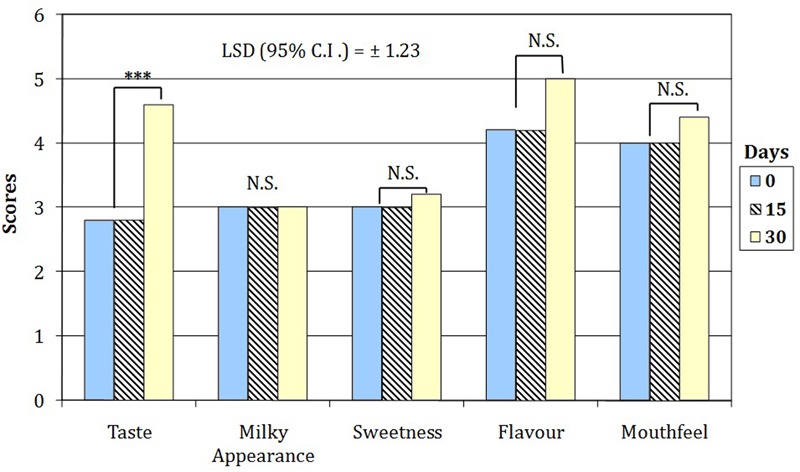
**Sensory attributes of WPF beverages fermented by bifidobacteria throughout the cold storage.** Scores attributed with a 5-point just about right (JAR) scale (1 = too weak, 3 = just about right; 5 = too strong). NS, values are not significantly different; ^∗∗∗^values were found significanlty different for *P* < 0.01.

Sensory evaluation showed the overall acceptability decreased from 7.8 to 4.6 and from 6.8 to 4.2 for WPF with inulin and RS, respectively. As concerns sensory descriptors, the three-way interaction between beverage, time of storage and sensory attributes was not statistically significant (*p* = 0.657); thus, the scores of attributes changed only in relation to the beverage and throughout the time of cold storage. Therefore, in order to examine the effect of the attributes in relations to the time on sensory score only two-way ANOVA analyses were statistically allowed (*P* < 0.00001) for each beverage. Indeed, the simple main effect analysis showed the sensory scores of the beverage M115 were significantly (*P* < 0.00001) higher than those registered by BBR8 only in relation to the mouthfeel notes (data not shown). By contrast, both beverages showed, on average, a highly significant (*P* < 0.01) increase in the taste score only after 30 days of cold storage (**Figure 4**).

The overall acceptability of WPF beverages, at the early stage of cold storage, showed values similar to those previously found in a probiotic yogurt containing skim milk powder and WPC (Antunes et al., 2005). The worsening in sensory attributes was previously found in a yogurt containing microencapsulated bifidobacteria cells (Kailasapathy, 2006). The sensory scores registered for the WPF beverages including RS and fermented with the M115 strain could be partially attributed to the positive influence of RS in accordance with results of Kailasapathy (2006) suggested as positive effect of capsulant and filler materials (alginate and Hi-Maize^TM^ Starch) in masking the grittiness (mouthfeel) of the yogurt fermented with bifidobacteria.

## Conclusion

The present work demonstrates that a whey-based substrate sustains the growth of bifidobacteria monocultures allowing to obtain a fermented beverage containing 20 g L^-1^ of whey proteins that are rich in branched chain and sulfur-containing amino acids.

The supplementation with prebiotic fibers improves the growth rate of some *Bifidobacterium* strains leading to set up a synbiotic food. In addition, after fermentation, the strains assayed resulted able to survive in these beverages under cold storage without being freeze dried, spray dried, or microencapsulated (McMaster and Kokott, 2005; Kailasapathy, 2006; Yeung et al., 2016).

Under the experimental conditions used in this study, the best result was obtained fermenting a whey protein medium, made up with 20 g L^-1^ of whey proteins and 10 g L^-1^ of RS, with the *B. pseudocatenulatum* M115. Further work will be addressed to improve sensory attributes of this new synbiotic whey protein fortified beverage.

## Author Contributions

SdC carried out experiments and evaluated viable cell loads of all Bifidobacteria strains; LQ evaluated dietary fibers and protein content throughout fermentation and cold storage; LC was responsible for sensory evaluation and carried out statistical analysis for all experiments; FDL cooperated with SdC in particular for experiment related to the M115 strain; FB being the lead investigator, designed the study and supervised the research team, drafted the manuscript and final proofreading. All authors contributed to the manuscript draft.

## Conflict of Interest Statement

The authors declare that the research was conducted in the absence of any commercial or financial relationships that could be construed as a potential conflict of interest.
